# Uncovering a unique pathogenic mechanism of SARS-CoV-2 omicron variant: selective induction of cellular senescence

**DOI:** 10.18632/aging.205297

**Published:** 2023-12-12

**Authors:** Franziska Hornung, Nilay Köse-Vogel, Claude Jourdan Le Saux, Antje Häder, Lea Herrmann, Luise Schulz, Lukáš Radosa, Thurid Lauf, Tim Sandhaus, Patrick Samson, Torsten Doenst, Daniel Wittschieber, Gita Mall, Bettina Löffler, Stefanie Deinhardt-Emmer

**Affiliations:** 1 Institute of Medical Microbiology, Jena University Hospital, Jena 07747, Germany; 2 Member of the Leibniz Centre for Photonics in Infection Research (LPI), Jena 07747, Germany; 3 Medicine/Pulmonary and Critical Care Division, University of California San Francisco, San Francisco, CA 94110, USA; 4 Else Kröner Graduate School for Medical Students “JSAM” Jena University Hospital, Jena 07747, Germany; 5 Klinik für Herz- und Thoraxchirurgie, Jena 07747, Germany; 6 Institute of Forensic Medicine, Jena University Hospital, Jena 07747, Germany; 7 Institute of Forensic Medicine, University Hospital Bonn, University of Bonn, Bonn 53111, Germany

**Keywords:** SARS-CoV-2, variant of concern, cellular senescence, lung airway cells

## Abstract

Background: SARS-CoV-2 variants are constantly emerging with a variety of changes in the conformation of the spike protein, resulting in alterations of virus entry mechanisms. Solely omicron variants use the endosomal clathrin-mediated entry. Here, we investigate the influence of defined altered spike formations to study their impact on premature cellular senescence.

Methods: In our study, *in vitro* infections of SARS-CoV-2 variants delta (B.1.617.2) and omicron (B.1.1.529) were analyzed by using human primary small alveolar epithelial cells and human *ex vivo* lung slices. We confirmed cellular senescence in human lungs of COVID-19 patients. Hence, global gene expression patterns of infected human primary alveolar epithelial cells were identified via mRNA sequencing.

Results: Solely omicron variants of SARS-CoV-2 influenced the expression of cell cycle genes, highlighted by an increased p21 expression in human primary lung cells and human *ex vivo* lungs. Additionally, an upregulated senescence-associated secretory phenotype (SASP) was detected. Transcriptomic data indicate an increased gene expression of p16, and p38 in omicron-infected lung cells.

Conclusions: Significant changes due to different SARS-CoV-2 infections in human primary alveolar epithelial cells with an overall impact on premature aging could be identified. A substantially different cellular response with an upregulation of cell cycle, inflammation- and integrin-associated pathways in omicron infected cells indicates premature cellular senescence.

## INTRODUCTION

Virus mutations of the severe acute respiratory syndrome coronavirus 2 (SARS-CoV-2) resulted in the occurrence of variants of concern (VOC), e.g., alpha (B.1.1.7), delta (B.1.617.2) and omicron (B.1.1.529) [1, 2]. Mainly mutations in genes encoding for the spike (S) protein could be detected, which facilitates the virus entry into the cell [3, 4].

The entry of enveloped viruses occurs via endocytosis or fusion with the plasma membrane. With its additional unique mutations, omicron variant favors the endosomal entry via clathrin-mediated endocytosis (CME) in contrast to the delta variant [4–6]. For this, the internalization of integrins plays a considerable role [7, 8]. Recently, it has been reported that integrin activation is a necessary step for SARS-CoV-2 infection [9].

Interestingly, recent studies point out that altered molecular regulation in endocytic pathways contributes to cellular senescence [10]. For instance, persistent activation of integrins due to downregulated CME leads to an increase in senescence-related gene expression [10]. In fact, SARS-CoV-2 already have been shown to induce senescence *in vitro* and *in vivo* with COVID-19 patients [11]. Cellular senescence is a stress response mechanism that is characterized by irreversible cell cycle arrest, altered metabolism, altered morphology, and the senescence-associated secretory phenotype (SASP) [12].

Although aging is one of the major risk factors for coronavirus disease 2019 (COVID-19), the role of SARS-CoV-2 infection on the establishment of cellular senescence is not yet clearly defined. We investigated cellular effects of delta and omicron variants *in vitro, ex vivo,* and in human lungs. We were able to identify that solely the omicron variant leads to premature cellular senescence.

## RESULTS

### SARS-CoV-2 omicron variant induce cellular senescence

**Figure 1:**
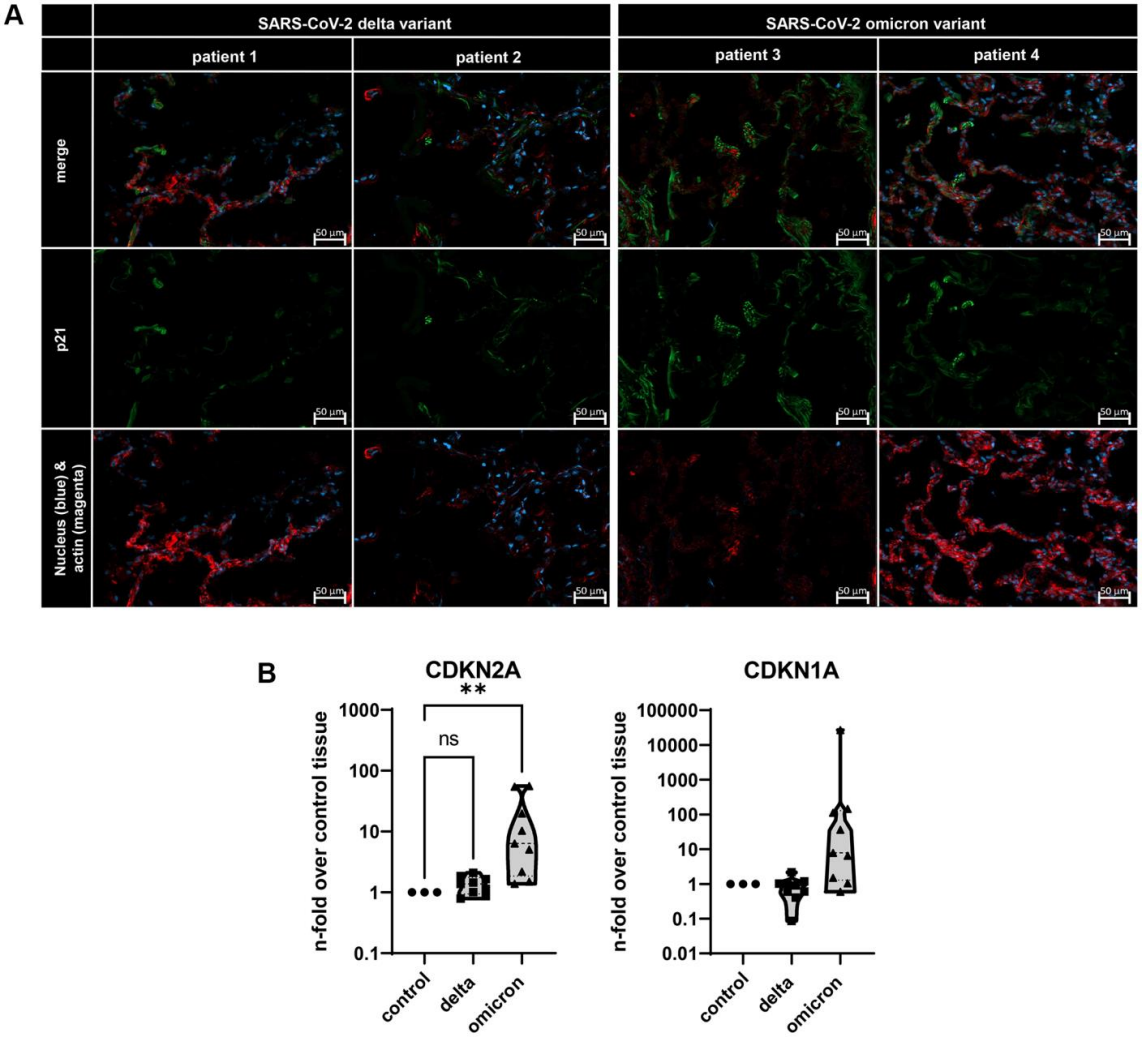
(**A**) Immunofluorescence staining of lungs from deceased COVID-19 patients. Lung tissue was stained with p21 (green), actin (red), and nuclei (blue) staining. Scalebar indicates 50 μm. (**B**) Gene expression of senescence marker CDKN1A and CDKN2A in three distinct lung biopsies of three deceased patients per SARS-CoV-2 variant. Expression levels are displayed normalized to healthy lung tissue from healthy lung transplantation tissue. P calculated by ANOVA with Kruskal-Wallis multiple comparisons tests (**B**), ^**^p < 0.05.

To identify the impact of two distinct entry mechanisms previously described, namely the membrane fusion of the delta variant and the CME of omicron, we analyzed the lungs of deceased COVID-19 patients (Figure 1) [3, 4]. For this, the lungs of two delta-variant infected patients and the lungs of two omicron variant infected patients were investigated by using immunofluorescence staining (Figure 1A). Here, a positive signal for p21 was confirmed for both variants. Notably, a stronger signal was identified for the omicron variant (Figure 1A). To quantify these qualitative results, we assessed the gene expression levels of both CDKN2A (p16) and CDKN1A (p21) in lung biopsies from three deceased patients per virus variant. Once more, infection with the omicron variant resulted in a significant upregulation of CDKN2A in lung tissue when normalized to uninfected control tissue. Additionally, the expression level of CDKN1A was also upregulated, although it did not reach statistical significance (Figure 1B).

**Figure 2:**
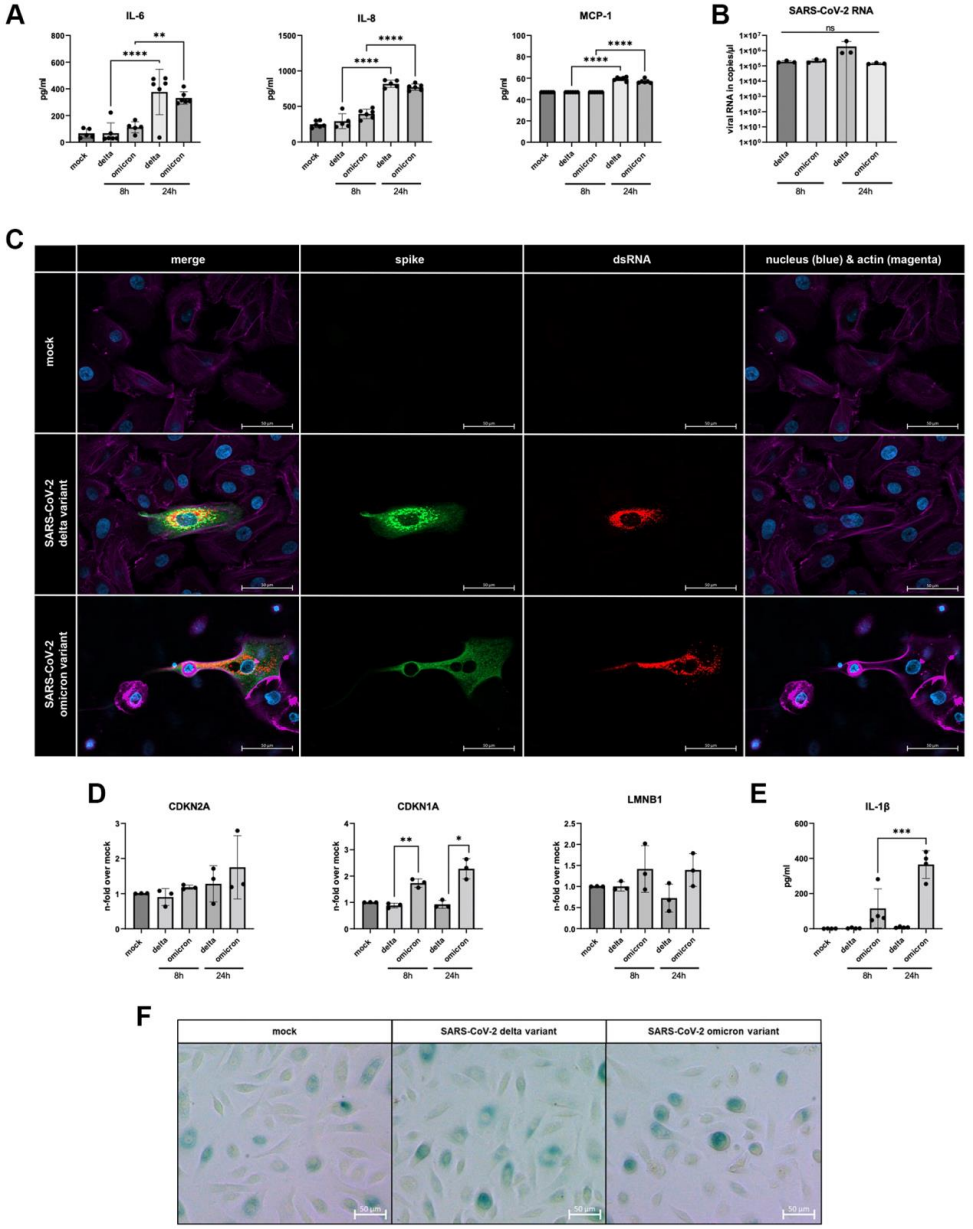
(**A**) Increased levels of IL-6, IL-8, and MCP-1 of infected supernatants of SAECs (*n* = 3) determined 8 h and 24 h p.i. compared to non-infected mock. (**B**) SAECs were infected with SARS-CoV-2 delta or omicron variant with an MOI of 5 for 24 h. Infected cells show clear signals for intracellular spike protein (green) and dsRNA (red). (**C**) SARS-CoV-2 RNA detected in cell lysates of infected SAECs after 8 h and 24 h p.i.. (**D**) Gene expression of senescence markers CDKN1A, CDKN2A, and LMNB1, normalized to mock cells. CDKN1A is significantly upregulated in cells infected with omicron variant compared to the delta variant. (**E**) Levels of the senescence-associated secretory phenotype (SASP) protein IL-1β are significantly increasing in omicron-infected cells from 8 h to 24 h p.i.. (**F**) β-Galactosidase staining of SAECs after 24 h p.i. with SARS-CoV-2. Scalebar indicates 100 μm. P calculated by one-way ANOVA with Tukey’s multiple comparisons tests (**A**, **E**) and Welch’s *t*-test (**D**), ^*^p < 0.01, ^**^p < 0.05, ^***^p < 0.001, ^****^p < 0.0001.

For the investigation of cell cycle components, we infected small alveolar epithelial cells (SAECs) with both SARS-CoV-2 variants. Here, we determined the inflammatory response after 8 h and 24 h of infection (Figure 2A). After 24 h of infection, a significant increase of IL-6, IL-8 and monocyte chemoattractant protein (MCP)-1 in both variants could be observed. In addition, both virus variants show the same ability to establish an infection (Figure 2B). The immunofluorescent staining of the infected cells revealed positive signals for the spike protein as well as the double-stranded RNA within the cytoplasm (Figure 2C).

Cellular senescence was analyzed once more via the determining the gene expression profile. A significant increase in CDKN1A mRNA was detected. The other analyzed marker CDKN2A and LMNB1 did not exhibit equally distinct results. Nevertheless, LMNB1 displayed a similar trend, with the highest average expression in omicron-infected cells (Figure 2D). Notably, the SASP-associated inflammatory protein IL-1β was detectable exclusively in omicron-infected cells (Figure 2E). Additionally, β-Galactosidase was stained in SAECs 24 h p.i. (Figure 2F). Upon examination, is important to note that it does not provide a clear qualitative difference between uninfected and both SARS-CoV-2 variants. However, it is worth mentioning that we did observe partially darker cells in infected samples. Moreover, there was an alteration in cell shape of SAECs infected with SARS-CoV-2 omicron (Figure 2F).

### mRNA sequencing indicates influence of the cell cycle

**Figure 3:**
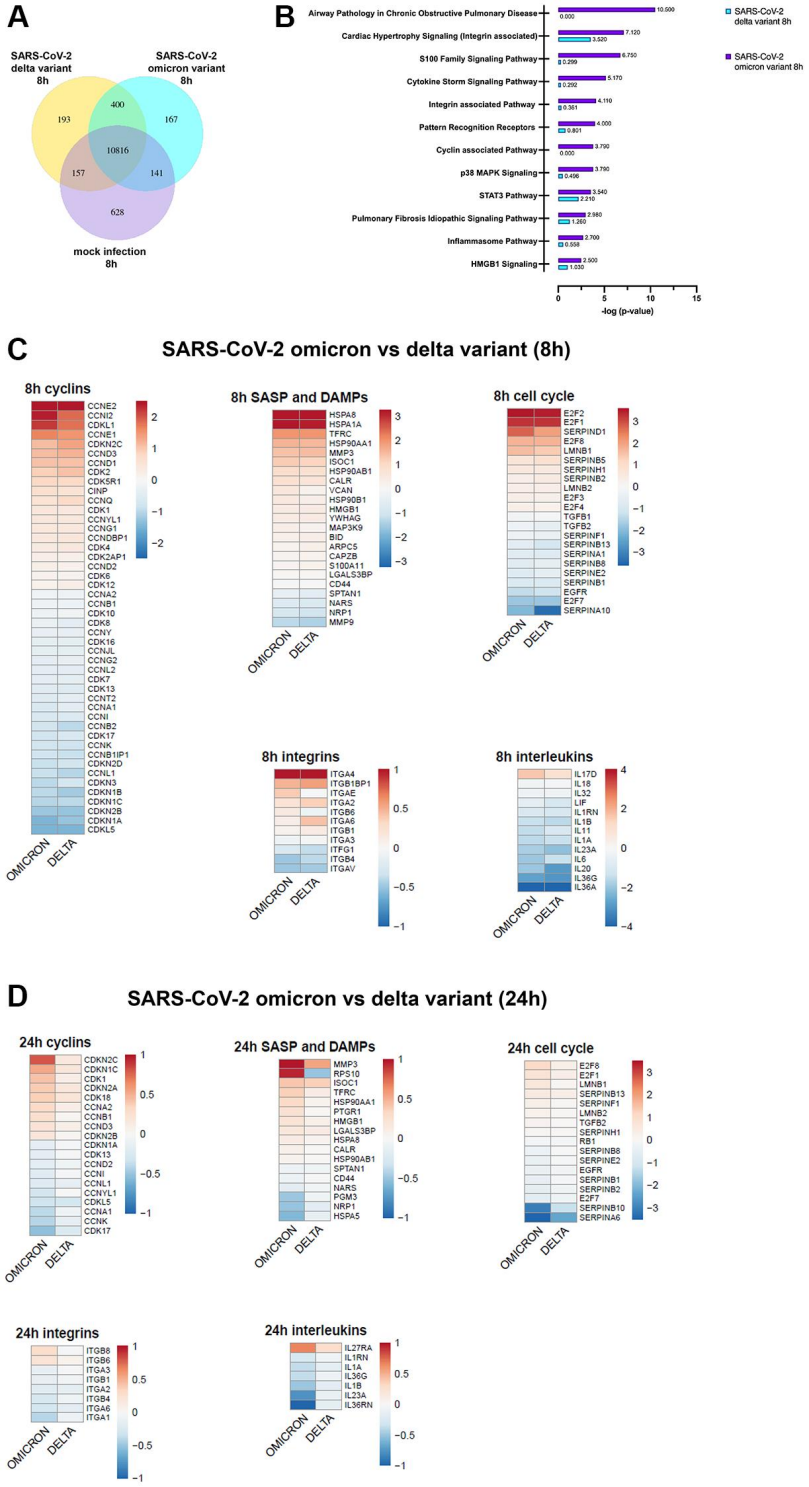
(**A**) Infection with SARS-CoV-2 variants alters the gene expression patterns of SAEC cells. Co-expression Venn Diagram showing regulated genes of mock, omicron- and delta variant infected cells identified via mRNA-sequencing 8 h p.i.. (**B**) Pathways with significant enrichment scores (-log (*p*-value)) revealed by QIAGEN Ingenuity Pathway Analysis 8 h p.i. indicates significant increase of gene regulation in omicron-infected cells. (**C**, **D**) Representative heatmaps of genes regulated due to omicron variant or delta variant, after 8 h (**C**) or 24 h (**D**) p.i. shown in relation to pathway or gene families. Here, selective genes for cyclins, senescence-associated secretory phenotype (SASP), Damage-associated molecular patterns (DAMPS), integrins, interleukins, and cell cycle are shown.

A genome-wide transcriptome analysis unveiled a unique expression of genes after 8 h (Figure 3A, Supplementary Figure 1). An ingenuity pathway analysis (IPA) of differentially regulated genes after 8 h revealed significant upregulation of cell cycle, inflammation- and integrin-associated pathways in omicron infected cells (Figure 3B).

Infection with SARS-CoV-2 affected the expression of cyclins, MAPK signaling genes, integrins, and cell cycle related genes (Figure 3C, 3D). Interestingly, after 24 h of omicron infection significant modifications of cell cycle related genes could be detected (Figure 3D). Cell cycle arrest was confirmed by the upregulation of p16 (CDKN2A), E2F1 and E2F8 after 24 h post infection with omicron (Figure 3D). MMP3 and HMGB1 were increased by both variants at both timepoints (Figure 3C, 3D). Interestingly, this rise was persistent in omicron variant and amplified even more after 24 h. (Figure 3D).

### Induction of cellular senescence in human *ex vivo* lung model

**Figure 4:**
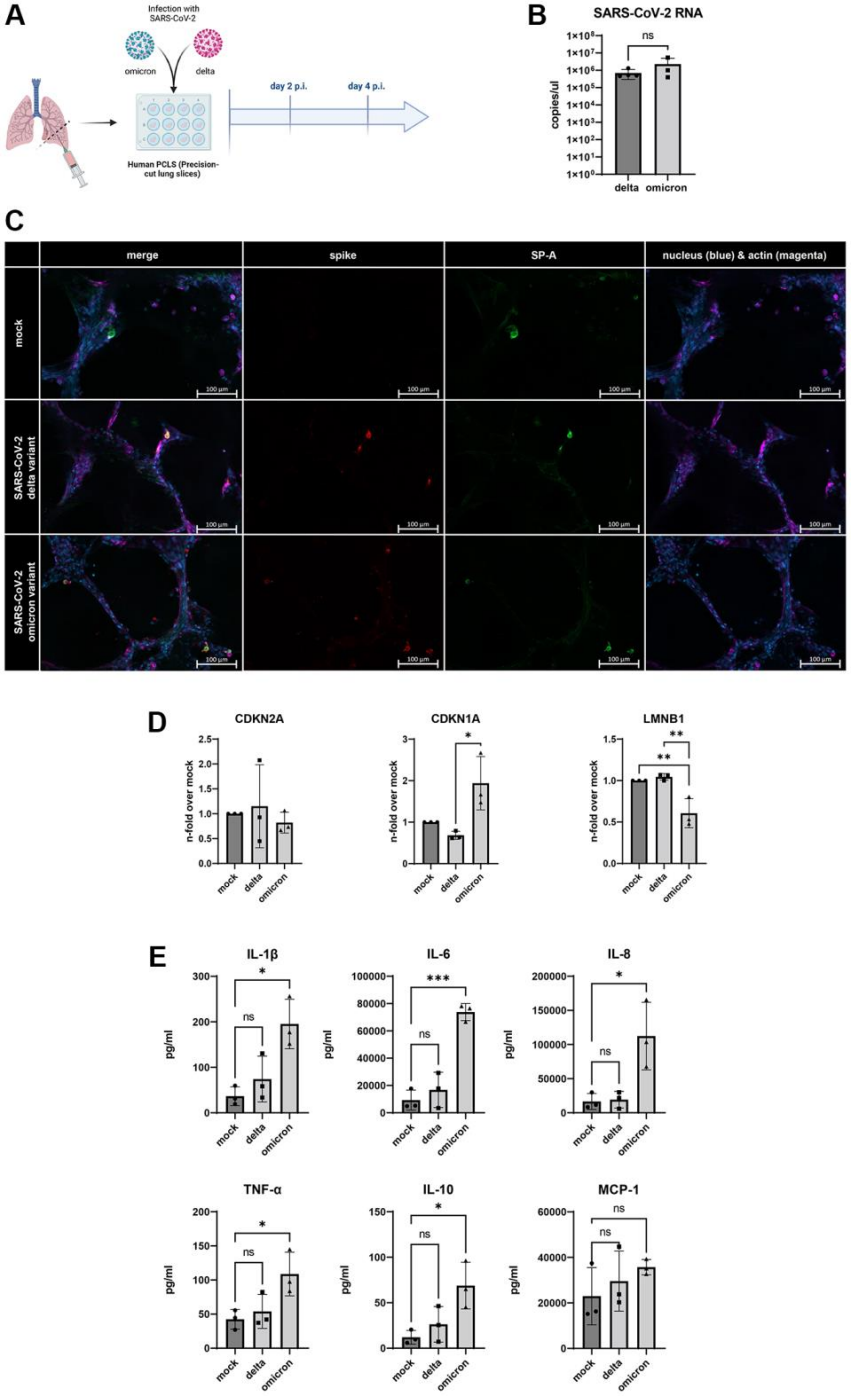
(**A**) Schematic overview of the experimental setup for the infection of human PCLSs with SARS-CoV-2 delta and omicron variant. Created with https://www.biorender.com/. (**B**) SARS-CoV-2 RNA detected in supernatants of infected human PCLSs after 2 d p.i.. (**C**) Immunofluorescent staining of mock and infected PCLS. Surfactant protein a (SP-A, green) positive cells are visible in the alveoli of mock and infected slices 4 d p.i.. Colocalization of spike protein (red) and SP-A positive cells in the alveolus of infected slices. Scalebar indicates 100 μm. (**D**) Gene expression of senescence markers CDKN1A, CDKN2A, and LMNB1, normalized to mock PCLSs. CDKN1A is significantly upregulated in cells infected with omicron variant compared to delta. LMNB1 expression is significantly downregulated in omicron slices compared to delta and mock (**E**) Levels of the senescence-associated secretory phenotype (SASP) proteins IL-1β, IL-6, IL-8, TNF-α, IL-10 are significantly upregulated in omicron- and delta-infected PCLSs at 2 d p.i. MCP-1 levels are on average highest in omicron-infected slices. *P* calculated by one-way ANOVA with Multiple comparisons (**D**, **E**), ^*^p < 0.01, ^**^p < 0.05, ^***^p < 0.001.

To verify our findings, we used a human *ex vivo* lung model (Figure 4A, Supplementary Figure 2). For this, human lungs slices were infected with SARS-CoV-2 delta and omicron variant for 4 d p.i.. Both variants show viral replication detectable by viral RNA in the supernatant, determined by qRT-PCR without significant differences between virus variants (Figure 4B). Additionally, immunofluorescence staining revealed positive signal for surfactant A protein producing cells, showing signs of SARS-CoV-2 infection by staining the spike protein (Figure 4C).

The determination of mRNA-expression shows an upregulation of parameters of cellular senescence. Interestingly, solely omicron-infected slices indicate an increased expression of CDKN1A with a decreased expression of LMNB1 (Figure 4D). Additionally, the analysis of SAPS-related inflammatory cytokines and chemokines in the supernatant of the infected lung slices revealed that infection with the omicron variant exclusively triggered an increased secretion of IL-1b, IL-6, IL-8, TNF-α, and IL-1β. While values for MCP-1 were on average highest in omicron slices, they did not reach statistical significance (Figure 4E).

## DISCUSSION

**Figure 5:**
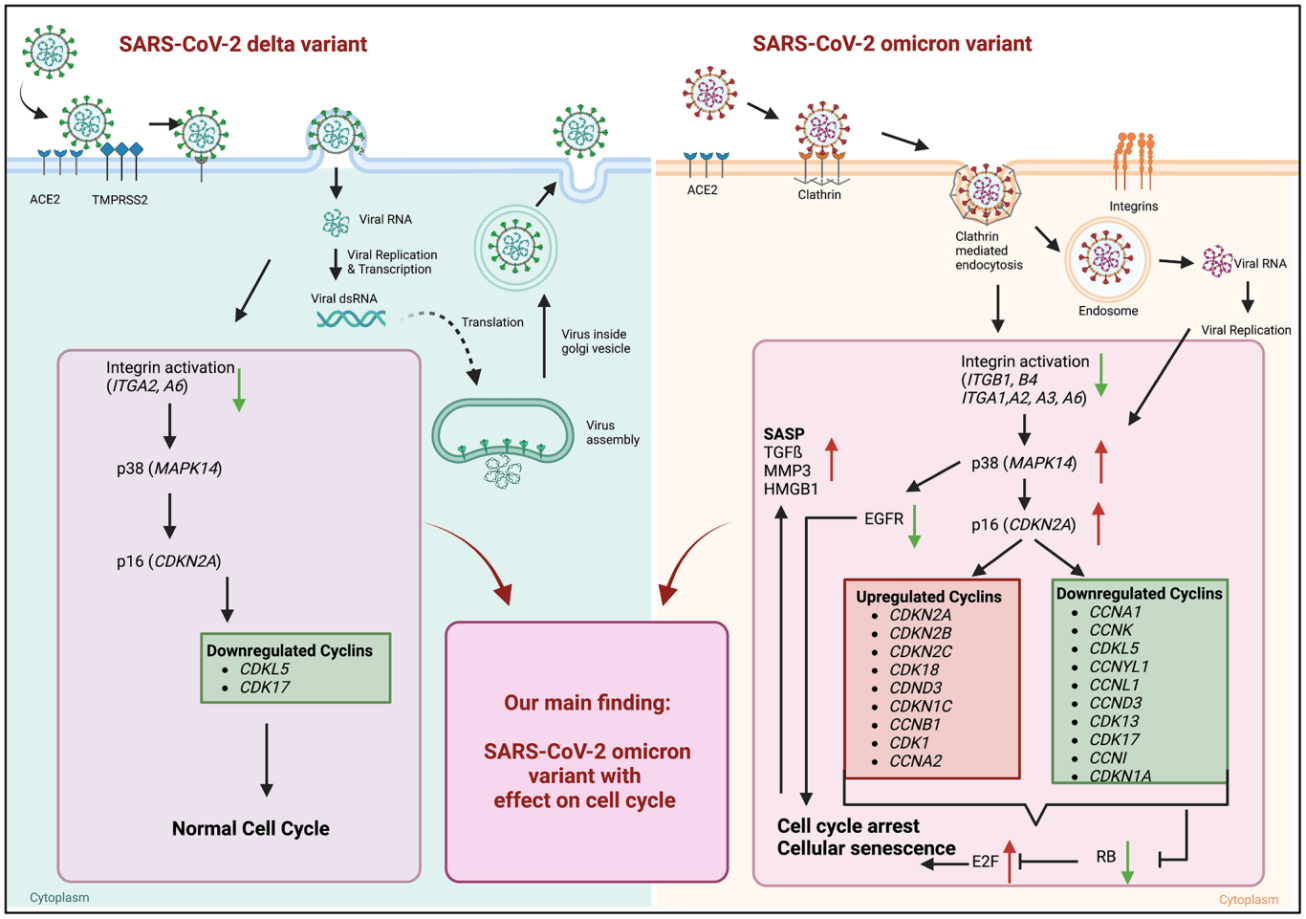
**Graphical abstract of known differences of SARS-CoV-2 delta and omicron variant entry and findings of our study obtained from mRNA sequence data of 24 h post infection.** Schematic overview of entry mechanisms of SARS-CoV-2 delta and omicron variants [5]. Delta variant (right panel) uses cell surface entry, by ACE2 and the protease TMPRSS2. Own data indicate a downregulation of the integrin activation without affecting p38 and p16 expression resulting in normal cell cycle. Omicron variant (right panel) prefers to use the clathrin-mediated endocytosis (CME) and cathepsin L as protease. Our results suggest the expression and activation of integrins (ITGB1, ITGB4, ITGA1, ITGA2, ITGA3, ITGA6) resulting an increase in p38 and p16. That increase in central kinases affects several cyclins, which in turn downregulates the retinoblastoma, increases the E2F transcription factors and results in cell cycle arrest and cellular senescence. Additionally, these changes lead to an increase in senescence-associated secretory phenotype (SASP). Thus, our findings indicate an influence of the altered cell entry mechanism of the omicron variant on the cell cycle. Created with https://www.BioRender.com.

Viral infections contribute to the pathogenesis of premature aging [13]. In previous work we investigated the effects of influenza A virus infection and the impact on cellular senescence following an acute infection [14]. However, despite the here reported paracrine effects, it remains unclear whether SARS-CoV-2 virus variants have the potential to induce cellular senescence and the mechanisms involved. Several studies have highlighted the occurrence of cellular senescence in COVID-19 infections. For instance, Nehme et al. in 2020 discussed the potential role of cellular senescence as a contributor to COVID-19 pathogenesis [15]. Our study provides insights in virus induced cellular senescence by using the SARS-CoV-2 delta and omicron variant. Both variants differ in their entry mechanism, leading to the activation of distinct intracellular signaling pathways (Figure 5). For the omicron variant, up to 60 mutations have been identified compared to the delta variant and the host-entry process has undergone a considerable modification [3].

Our results indicate a robust infection of epithelial cells *in vitro* and specifically of type 2 alveolar cells in a complex human *ex vivo* model by both variants. However, only the omicron variant promoted cellular senescence in SAECs and in *ex vivo* infected human lung slices. Interestingly, both cellular senescence and SARS-CoV-2 infection exhibit a similar pro-inflammatory phenotype. This association is further supported by the concept of inflammaging, which proposes that the heightened levels of local and systemic pro-inflammatory cytokines associated with aging play a crucial role in the development of the cytokine storm observed during COVID-19 [16]. Our findings align with this concept, as have observed a combination of cell cycle arrest, increasing levels of SASP factors in response to the SARS-CoV-2 infection.

We confirmed that solely omicron-infected cells were in irreversible cell cycle arrest, displaying an increased expression of SASP factors and altered integrin-associated pathways [9, 17]. Transcriptome analysis of omicron-infected SAECs at 24 hours p.i. revealed upregulated expression of senescence associated factors p16, p21, p38, E2F1 and E2F8 genes. These results were verified in human lung tissue from deceased COVID-19 patients, indicating a higher p21 immunofluorescence signal in omicron histological samples and increased expression of CDKN2A in extracted RNA from lung biopsies (Figure 1). Our findings are consistent with existing literature suggesting that SARS-CoV-2 induce senescence [11]. However, this study emphasizes that the omicron variant, in particular, leads to this senescent phenotype.

Omicron is an immune escape variant with an altered cell entry leading to high transmission rates [18]. In terms of clinical outcomes and disease severity, the presence of this prominent senescent phenotype does not align with the observed clinical presentation. While the core symptoms of COVID-19 remain largely consistent, the omicron variant has presented with some distinct differences. Here, milder respiratory symptoms with less involvement of the lower respiratory tract were prevalent [19]. One of the main differences between omicron and delta is the severity of illness. In general, omicron has been associated with a lower risk of severe disease and hospitalization. This has been attributed to a combination of factors, including potential changes in the virus’s spike protein and a higher level of pre-existing immunity in the population due to prior infection or vaccination. On the other hand, delta infections were more likely to result in severe respiratory symptoms and hospital admissions.

Comparisons of long-term sequelae due to an infection with SARS-CoV-2 detect a relationship between the severity of infection and the occurrence of Long-COVID [20]. However, other studies suggest that the burden of post-covid complaints is similar for omicron and delta [21].

Stewart et al. 2023 postulates that COVID-19 may lead to progressive lung damage with lung fibrosis [22]. A distinct morphological pattern of a COVID-19-fibrosis is suggested by Kamp et al. 2023 [23]. However, most clinical studies unfortunately do not address the difference between omicron and delta infections. Whereas a virus-related involvement in the pathogenesis of pulmonary fibrosis has been reported in the literature [24].

Infections with SARS-CoV-2 have been associated with the induction of cellular senescence within the lung. Our findings suggest that the omicron variant, in particular, leads to premature senescence in *in vitro*, *ex vivo*, and in lung tissue models. This difference may be attributed to the distinct endocytic cell entry and intracellular pathways of the omicron variant when compared to the delta variant. The induction of cellular senescence in lung tissue following acute SARS-CoV-2 infection could potentially contribute to the reported cytokine storm and the development of long-COVID.

## METHODS

### Virus strains

SARS-CoV-2 delta variant was isolated from patient material (SARS-CoV-2/human/DEU/vi0114749/2021) (Genbank: ON650061.1) and cultivated as previously described [25]. Omicron variant (human, 2021, Germany, B.1.1.529) was received from the European Virus Archive (EvaG).

### COVID-19 patient autopsy

Autopsies were approved by the local ethics board as described previously [26]. Patient 1, 2, and 3 died after COVID-19 infection with the SARS-CoV-2 delta variant, Patient 4, 5, and 6 after omicron variant infection (Supplementary Table 1). The shock-frozen lung tissue samples further processed for qRT-PCR and cut with a microtome (Leica, CM1950) (10 μm) and stored on microscopical slides until staining at −20°C.

### *In vitro* infections

Human-derived small airway epithelial cells (SAEC, Lonza, Switzerland) were incubated with a multiplicity of infection (MOI) of 5 of the SARS-CoV-2 variants diluted in SAEC Cultivation Medium (Lonza) supplemented with 1% penicillin/streptomycin (P/S, Lonza, Basel, Schweiz) for 1 h at 37°C, 5% CO2. After one washing step, the cells were incubated in fresh medium for 8 h, 1, and 3 days.

### Human *ex vivo* PCLS (precision-cut lung slices)

Human lung lobe specimens were received from the Department of Cardiothoracic Surgery, Jena University Hospital-Friedrich Schiller University of Jena, approved by the local ethics board (no. 2018-1263, 2020-1894, and no. 2020-1773).

The tip of the human lung lobe (5–10 cm^3^) was stored in cold DPBS between 4–10 hours at 4°C. The main bronchus was cannulated with 50 ml original perfusion syringe (BBraun, Germany). The small bronchioles were closed by surgical clamps or by superglue to avoid agarose leaks. 4% top vision low-melting point agarose (Thermo Fisher Scientific Baltics, Lithuania) was prepared in distilled sterile water, cooled down to 45°C and mixed with 37°C warm DMEM/F12 w. o. phenol-red (Gibco, Thermo Fisher Scientific, Germany) at a 1:1 ratio. 50 to 100 ml of this agarose mixture was instilled into the tissue and kept on ice for 30 min to solidify. Subsequently, tissue was sliced into 1–1.5 cm³ cubes and cut into 300 μm slices by Microtome (Leica – VT1200S, Leica Biosystems, Germany). Slices were stored in 12 well plates with 1 ml DMEM/F12 supplemented 1% P/S, at 37°C with 5% CO_2_ (Supplementary Figure 2).

After 48 hrs of adjustment to conditions in the well plate, slices were infected with a concentration of 5 x 105 PFU/ml of the SARS-CoV-2 variants diluted in cultivation medium for 2 hours at 37°C, 5% CO_2_. After one washing step, fresh medium was added and PCLSs were monitored for 2 and 4 days p.i..

### β-Galactosidase staining

To analyze the senescent phenotype in SAEC in response to infection with the SARS-CoV-2 variants, cells were stained with β-galactosidase staining kit (SA β-gal staining, Biovision, Cambridge, UK) according to the manufacturer’s instructions.

### Immunofluorescence staining

Lung slices from deceased patients were fixed in ice-cold acetone. Afterward, slides were incubated with the primary antibody against p21 (1:200, Invitrogen, Carlsbad, CA, USA) diluted in Dako antibody diluent (Dako) in a humidity chamber for 1 hour at RT. Subsequently, slides were incubated with the secondary antibody Alexa Fluor^®^ 488 AffiniPure Donkey Anti-Rabbit IgG (H+L, 1:500, Jackson Immuno Research, West Grove, PA, USA). Actin filaments were stained with BODIPY^®^ 558/568 phalloidin (Invitrogen) according to the manufacturer’s instructions.

For immunocytochemistry, cells were fixed in 4% paraformaldehyde (PFA, Sigma-Aldrich) for 30 min, 37°C. Cells were subsequently permeabilized with 0.1% Triton-X (Roth) for 30 min at RT, blocked with 3% bovine serum albumin (BSA, Roth) for 30 min at RT, and incubated with the primary antibodies against double-stranded RNA (dsRNA, 1:200, Jena Bioscience) and SARS-CoV-2 (COVID-19) Spike RBD (HL1014) (1:200, GeneTex) at 4°C overnight. Afterward, secondary antibodies Alexa Fluor^®^ 488 AffiniPure Goat Anti-Rabbit IgG (H+L, 1:500, Jackson ImmunoResearch, and Cy™3 AffiniPure Donkey Anti-Mouse IgG (H+L, 1:500, Jackson Immuno Research) were used. Actin filaments were stained with Alexa Fluor™ Plus 647 Phalloidin (Thermo Fisher Scientific) according to the manufacturer’s instructions.

PCLS were fixed with 4% PFA for 2 hrs at 37°C. Slices were permeabilized and blocked in the same manner as described for the mono-culture, yet for a prolonged time (1 hr). Slices were stained with the primary antibodies SARS-CoV-2 (COVID-19) Spike RBD (HL1014) (1:200, GeneTex) and surfactant protein A (SP-A, 1:100, Novus Biologicals) and the secondary antibodies Cy3-conjugated AffiniPure Donkey Anti-Rabbit IgG (H+L, 1:500, Jackson ImmunoResearch) and Alexa Fluor^®^ 488 AffiniPure Donkey Anti-Goat IgG (H+L, 1:500, Jackson Immuno Research). Actin filaments were again stained with Alexa Fluor™ Plus 647 Phalloidin.

Cells, as well as lung samples from patients, and human *ex vivo* lung slices, were mounted with DAPI Fluoromount-G (SouthernBiotech, USA) and analyzed with AxioObserver Z.1 Microscope (Zeiss, Germany) and the corresponding Software ZEN.blue 3.3.

### Cytokine determination

Cytokine analysis of supernatants from *in vitro* and *ex vivo* infection setups was performed with the LEGENDplex™ Human Inflammation Panel 1 (BioLegend, San Diego, CA, USA) according to manufacturer’s instructions. Samples were analyzed on the Accuri C6 Plus Cytometer (BD Biosciences, Heidelberg, Germany) and FACS Symphonie A1 (BD Biosciences).

### RNA-isolation and qRT PCR

RNA isolation from patient lung biopsies was carried out using the viral RNA easy Mini kit following tissue homogenization, as previously described [26]. We analyzed three distinct lung tissue biopsies from each COVID patient, while leftover tissue from a donor lung transplantation served as healthy control samples for the following PCR analysis.

RNA of *in vitro* experiments was extracted using the Qiagen RNeasy Mini Kit (QIAGEN, Hilden, Germany). RNA extraction of the *ex vivo* PCLS was performed with Trizol LS Reagent (Thermo Fisher Scientific).

cDNA synthesis was accomplished with the High-Capacity cDNA Reverse Transcription Kit (Thermo Fisher Scientific) was used. Maxima SYBR Green qPCR Master Mix (Thermo Fisher Scientific) and Rotor-Gene Q (Qiagen) were used for qRT-PCR with indicated primer pairs (Supplementary Table 2). Data are presented as n-fold over the corresponding control (mock). The primer sets used for qRT-PCR (95°C for 10 min, followed by 45 cycles of 95°C for 10 s, 60°C for 20 s, and 72°C for 30 s) were produced by metabion international AG (Planegg/Steinkirchen Germany, Supplementary Table 1).

Quantification of virus RNA copies was performed with the RIDA GENE SARS-CoV-2 Kit (r-biopharm, Germany) according to the manufacturer’s instruction.

### mRNA sequencing and pathway analysis

Preparation of RNA library and transcriptome sequencing of the SAEC cells was conducted by Novogene Co., LTD., (Beijing, China), performed on the Illumina platform Novaseq 6000 S4 flowcell V1.0, based on the mechanism of sequencing by synthesis (SBS) and the PE150 strategy (NEB Next Ultra RNA Library Prep Kit. R (v3.6.3) was used to generate heatmaps (R Foundation for Statistical Computing, https://www.R-project.org/). For pathway analysis, QIAGEN Ingenuity Pathway Analysis was performed.

### Statistical analysis and scheme design

Statistical analysis was carried out using GraphPad Prism 9. Illustrations were created with https://www.biorender.com/.

### Data availability statement

The data that support the findings of this study are available from the corresponding author upon reasonable request.

## Supplementary Materials

Supplementary Figures

Supplementary Tables
