# Supplementary material for: Uncovering a unique pathogenic mechanism of SARS-CoV-2 omicron variant: selective induction of cellular senescence
Source: Aging (Albany NY). 2023 Dec 12;15(23):13593–607. doi: 10.18632/aging.205297 (PMC10756098; doi:10.18632/aging.205297)
Supplement: Supplementary Figures [file aging-15-205297-s001.pdf]

SUPPLEMENTARY FIGURES

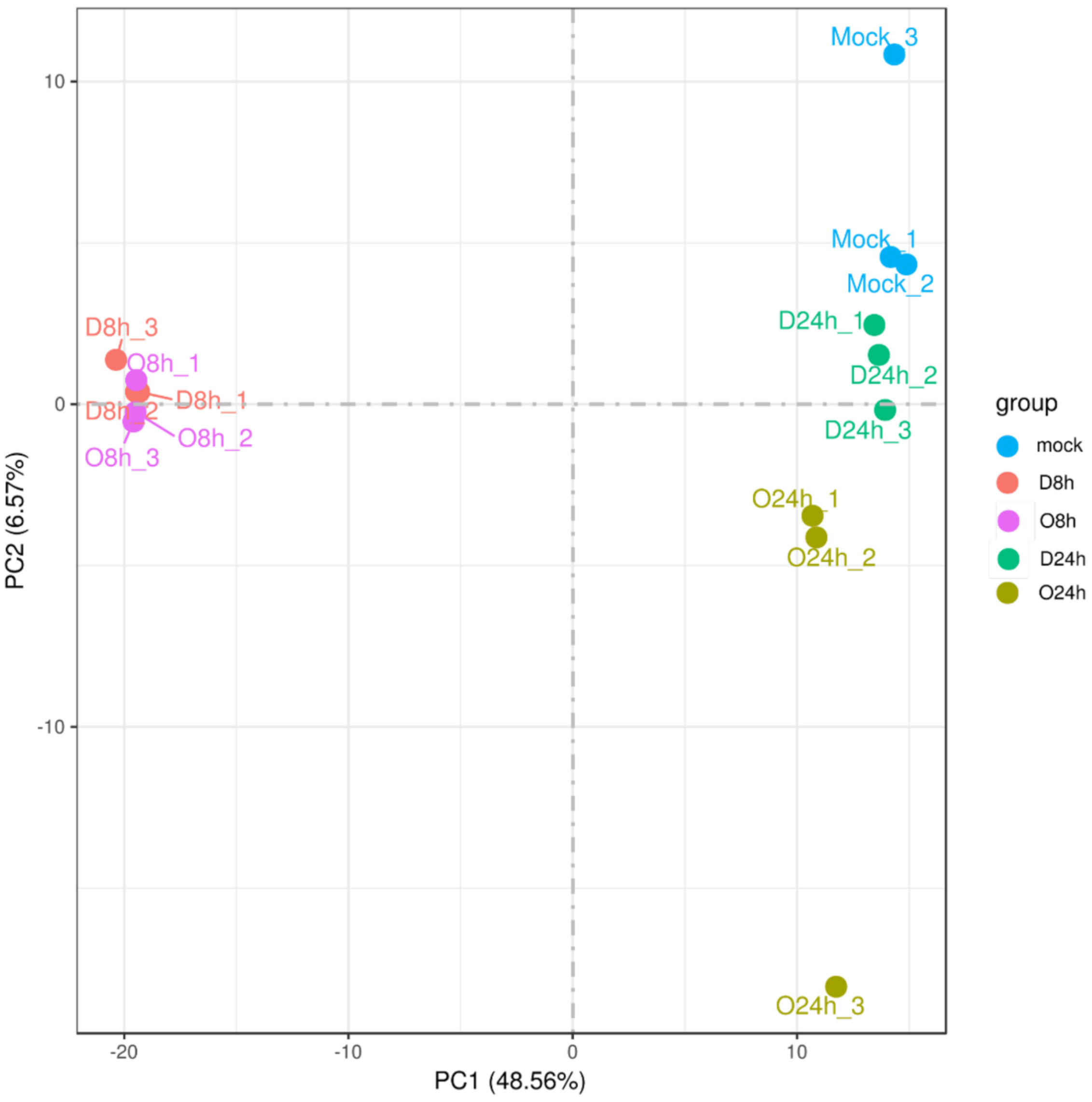

Supplementary Figure 1. Principal component analysis (PCA) shows discrete clusters undergoing different infections conditions of SAECs with omicron (O) or delta (D) infection for 8 h or 24 h and without (mock) infection.

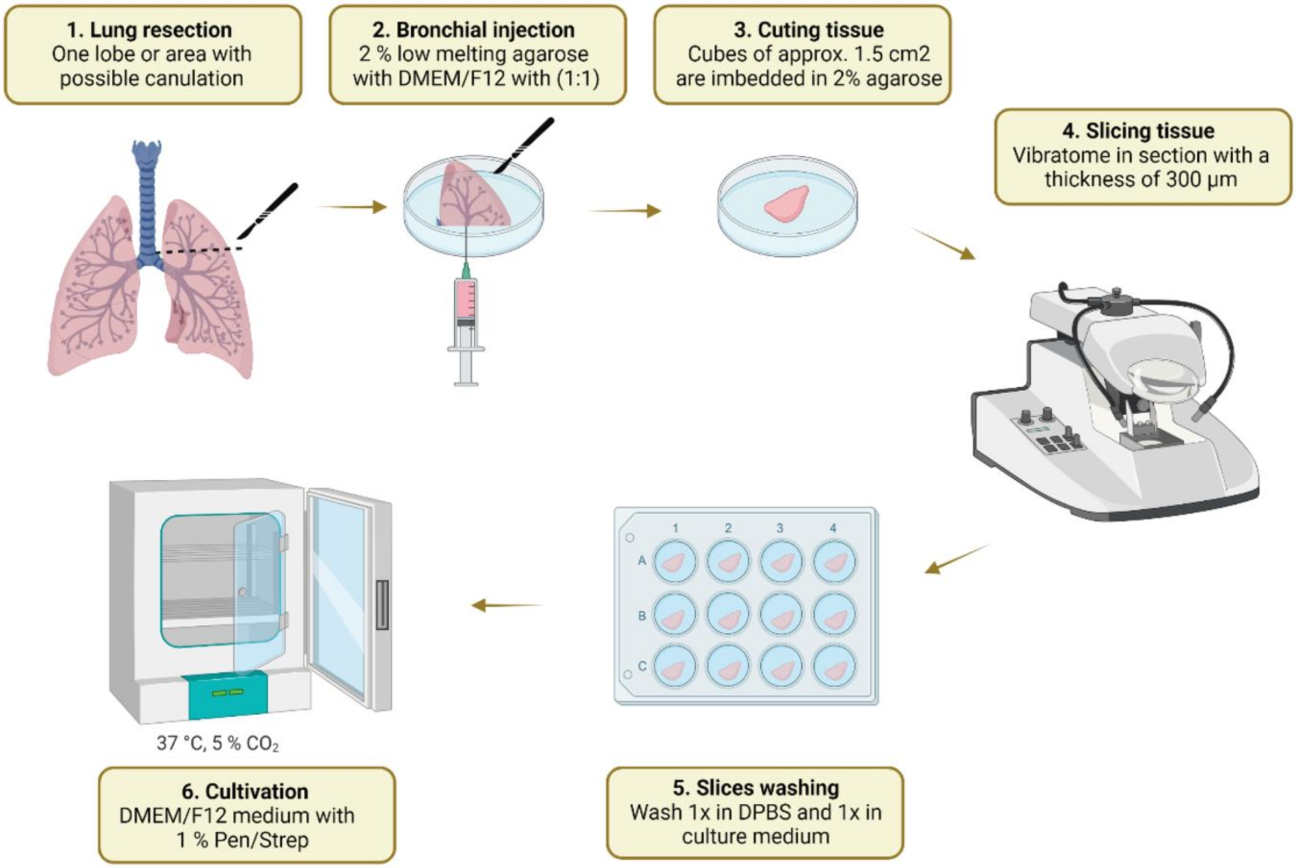

Supplementary Figure 2. Schematic overview of the experimental setup for the infection of human PCLSs, created with https://www.biorender.com/. After explanation of the lungs, agarose was injected into the bronchial system and cut into slices.
