# Supplementary material for: Uncovering a unique pathogenic mechanism of SARS-CoV-2 omicron variant: selective induction of cellular senescence
Source: Aging (Albany NY). 2023 Dec 12;15(23):13593–607. doi: 10.18632/aging.205297 (PMC10756098; doi:10.18632/aging.205297)
Supplement: Supplementary Tables [file aging-15-205297-s002.pdf]

SUPPLEMENTARY TABLES

Supplementary Table 1. Clinical characteristics of deceased patients infected with SARS-CoV-2 delta or omicron variant.

Patient	Age	Sex	SARS-CoV-2 variant	Cause of Death
1	78	male	delta	mutiple organ failure
2	54	male	delta	lung failure
3	64	male	delta	lung failure
4	63	male	omicron	paralytic ileus
5	64	male	omicron	mesenteric ischemia
6	35	female	omicron	cardiomyopathy

Supplementary Table 2. Overview of primer sets used for qRT-PCR (95°C for 10 min, followed by 45 cycles of 95°C for 10 s, 60°C for 20 s, and 72°C for 30 s) produced by metabion international AG (Planegg/Steinkirchen Germany).

Sequence name	Sequence
ACTB.Fl	CATGTACGTTGCTATCCAGGC
ACTB.Rl	CTCCTTAATGTCACGCACGAT
CDKN2A (p16)_F	CTCGTGCTGATGCTACTGAGGA
CDKN2A (p16)_R	GGTCGGCGCAGTTGGGCTCC
CDKN1A(p21)_F	TCACTGTCTTGTACCCTTGTGC
CDKN1A(p21)_R	GGCGTTTGGAGTGGTAGAAA
LMNBl_F	TTGGATGCTCTTGGGGTTC
LMNBl_R	AAGCAGCTGGAGTGGTTGTT
